# PANDA: AND logic-gated RNA sensing enabled by a photo-activated RCA-Argonaute cascade

**DOI:** 10.1039/d6sc00827e

**Published:** 2026-04-09

**Authors:** Xinxin Ke, Heming Fan, Jingwen Qu, Junlan Wang, Yilong Wang, Tengfei Xu, Chuanxia Chen, Chunyi Hu, Tao Hu

**Affiliations:** a National Clinical Research Center for Children and Adolescents' Health and Diseases, Children's Hospital, Zhejiang University School of Medicine Hangzhou 310058 China hutaozd@zju.edu.cn; b Department of Biological Sciences, Faculty of Science, National University of Singapore Singapore 117543 Singapore; c College of Pharmaceutical Sciences, Zhejiang University Hangzhou 310052 China; d School of Materials Science and Engineering, University of Jinan Jinan 250022 China

## Abstract

Achieving stable, multiplexed, and affordable nucleic acid detection in a true one-pot format remains a long-standing challenge for molecular diagnostics. Here, we report PANDA, a photo-activated rolling circle amplification (RCA)-Argonaute (Ago) cascade that encodes a stringent molecular AND logic gate between optical activation and target RNA recognition to enable highly sensitive, programmable RNA detection. In this system, ultraviolet (UV) irradiation triggers photolysis of the precursor probe, simultaneously generating a 5′-phosphorylated DNA guide and circularizable padlock templates, while target RNA selectively initiates ligation and RCA. Signal generation occurs only upon convergence of these two orthogonal inputs, enforcing strict dual-input gating prior to Ago activation. Once engaged, Ago catalyzes continuous guide regeneration and sequence-directed reporter cleavage, producing signal boosting outputs within a single reaction vessel. The modular architecture further enables parallel assembly of multiple AND logic gates synchronized by a common optical trigger yet paired with distinct RNA targets, allowing controllable multichannel detection without physical compartmentalization. By integrating optochemical control with RCA-Ago-mediated catalytic turnover, PANDA establishes a streamlined, stable, and cost-efficient framework for logic-gated nucleic acid diagnostics and multiplexed RNA sensing.

## Abstract 2

PANDA enables single-pot assembly of a synchronized molecular AND gate *via* modular photolabile probes, achieving multichannel RNA sensing with ultra-low background, precise temporal control, and minimal crosstalk.

## Graphical abstract

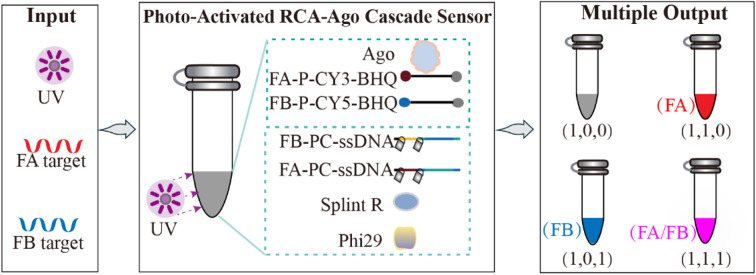

## Introduction

Nucleic acid testing underpins modern diagnostics by enabling direct interrogation of pathogen and host genetic information for early disease diagnosis, viral load monitoring, and mutation profiling.^1–4^ Conventional diagnostics remain dominated by the polymerase chain reaction (PCR), with real-time quantitative PCR (qPCR) as the clinical gold standard owing to its high sensitivity and broad dynamic range.^5–7^ However, the reliance on complex instrumentation and rigorously controlled laboratory environments restricts PCR-based assays largely to centralized settings. Recently, isothermal amplification approaches, including recombinase polymerase amplification (RPA), loop-mediated isothermal amplification (LAMP), and rolling circle amplification (RCA), have circumvented the need for thermal cycling and thus facilitated point-of-care deployment.^8–12^ Yet, these methods are prone to nonspecific amplification and background signal accumulation, which compromise assay reliability and multiplexed readout.

Clustered regularly interspaced short palindromic repeats (CRISPR)-based diagnostics have further advanced nucleic acid detection by coupling programmable sequence recognition with enzymatic signal transduction. CRISPR/Cas12, Cas13, Cas3, and related systems enable highly specific fluorescence or colorimetric readouts and have been widely adopted across pathogen detection and molecular profiling.^13–17^ Despite their conceptual elegance, most CRISPR assays rely on the nonspecific collateral cleavage of reporter substrates. This mode of signal generation inherently limits channel orthogonality in multiplex formats and often necessitates physical separation of reactions or elaborate assay designs to avoid crosstalk. In addition, the intrinsic instability of crRNAs complicates reagent storage and field deployment under non-ideal conditions. Photocleavable linkers have been incorporated into CRISPR systems to achieve light-controlled activation, typically by caging crRNAs or Cas proteins and restoring activity upon illumination.^18–20^ While these approaches provide temporal control over nuclease function, light primarily acts as an external switch, and signal generation still depends on RNA guides and collateral cleavage. The overall detection framework therefore remains governed by a single-trigger mechanism. These shortcomings highlight the need for alternative programmable biosensing mechanisms that can enforce strict target specificity and multiplexing capability without sacrificing simplicity.

Argonaute (Ago) proteins have emerged as a compelling alternative class of programmable nucleases capable of alleviating several of these limitations.^21,22^ Unlike CRISPR-Cas systems, many prokaryotic Agos are programmed by short single-stranded DNA guides (gDNAs), which are chemically robust, cost-effective to synthesize, and amenable to long-term storage without specialized protection.^23–26^ Notably, several Ago variants (*e.g.*, *Pyrococcus furiosus* Argonaute (*pf*Ago) and *Thermus thermophilus* Argonaute (*Tt*Ago)) retain robust catalytic activity at elevated temperatures, rendering them highly compatible with isothermal amplification workflows.^27–30^ By capitalizing on their distinct temperature activation windows, thermal gating provides a powerful strategy to temporally coordinate otherwise incompatible enzymatic modules within a single reaction vessel, enabling true one-pot operation without intermediate handling.

Notably, Ago nucleases directly cleave single-stranded nucleic acids with programmable specificity, providing a natural mechanistic complement to RCA. RCA generates long continuous ssDNA amplicons that are ideal substrates for repeated enzymatic cleavage and signal turnover. Together, these features position Ago-based platforms as promising engines for stable, multiplexed, and affordable nucleic acid diagnostics. In contrast to CRISPR effectors, Ago cleavage is strictly sequence-directed and does not produce nonspecific collateral activity, enabling channel-specific signal generation within a shared reaction environment. However, translating these biochemical advantages into a fully integrated, logic-controlled, and truly single-pot assay architecture capable of precise reaction coordination and signal routing remains an unmet challenge.

Here, we bridge this gap by developing a one-pot, photo-activated cascade assay, termed PANDA, that encodes a stringent molecular AND logic gate between optical activation and target RNA recognition. In this system, a brief UV illumination triggers photolysis of a caged DNA probe, simultaneously releasing a 5′-phosphorylated DNA guide and generating circularizable padlock templates, while the presence of target RNA selectively initiates ligation and RCA. Critically, signal generation occurs exclusively upon convergence of these two orthogonal inputs, enforcing strict dual-input gating before activation of the *pf*Ago nuclease. Functional guide DNA is generated only when optical activation and target-dependent amplification occur together, establishing a cascade-based logic control rather than simple photo-triggered activation. Once engaged, *pf*Ago drives continuous guide regeneration and sequence-programmed reporter cleavage, yielding enhanced fluorescence outputs within a single reaction vessel. Beyond a single logic gate, the modular design of photolabile scaffolds enables the parallel assembly of multiple AND logic gates, each synchronized by a common optical trigger yet paired with distinct RNA targets, thereby allowing concurrent, multichannel detection in a shared chemical environment. Although distinct fluorescent reporters are employed for different channels, signal specificity is maintained through guide-dependent Ago cleavage and dual-input gating, allowing simultaneous operation without physical separation.

By integrating optochemical control with Ago-driven catalytic turnover, PANDA delivers a streamlined, stable, and cost-efficient platform for programmable RNA detection that overcomes key bottlenecks of CRISPR-based diagnostics, including guide instability, cross-reactivity in multiplexing, and cumbersome multistep workflows. This work extends beyond enzyme substitution and establishes a photo-controlled cascade architecture that integrates optical input, target recognition, amplification, and sequence-specific cleavage within a unified one-pot system. This strategy provides a practical solution for sensitive and field-deployable molecular diagnostics, and introduces a generalizable framework for constructing logic-gated biomolecular circuits capable of coordinated, high-fidelity signal processing in complex sensing environments.

## Results and discussion

### Photolysis-driven AND logic gate in a sequential molecular cascade

**Fig. 1:**
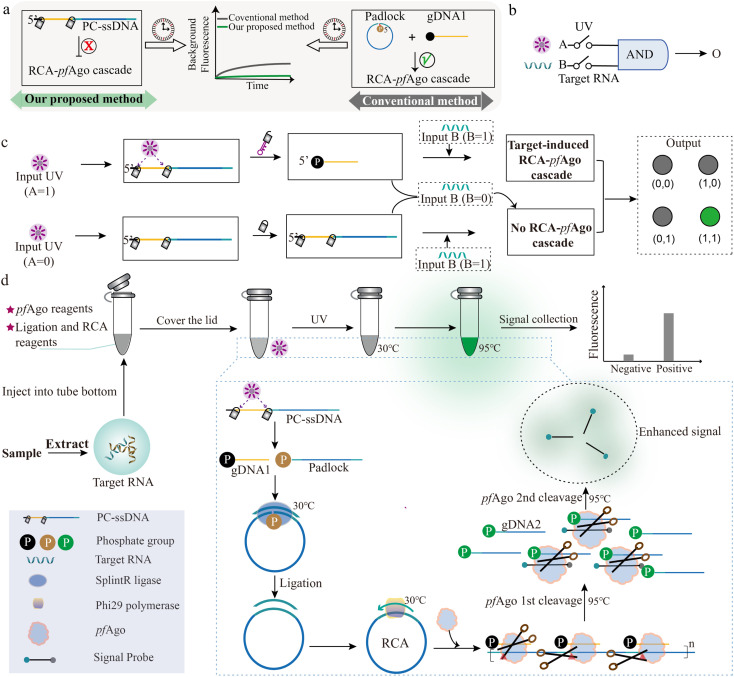
Photochemical triggering and target-dependent amplification converge to form a Boolean AND gate. (a) Conceptual comparison between the photo-gated PANDA strategy and the conventional RCA-Ago workflow. (b) Schematic abstraction of the AND logic gate, depicting UV irradiation (Input A) and target nucleic acid (Input B) as dual triggers required for output generation. (c) Mechanistic schematic of the AND-gated cascade showing photo-release of gDNA1 and the padlock and subsequent target-driven RCA-*pf*Ago signal generation. (d) Workflow diagram of the photo-activated AND-gated assay, showing sample nucleic acid introduction, illumination-driven probe activation, and isothermal RCA at 30 °C, followed by *pf*Ago activation at 95 °C to execute sequential cleavage events that yield the final fluorescent readout.

As shown in Fig. 1a, we designed a photo-gated DNA probe in which both the guide DNA and padlock sequences are covalently caged within a single photocleavable ssDNA (PC-ssDNA) *via* two photolabile linkers (Fig. S1). This design enforces a stringent “OFF” state prior to UV irradiation, effectively preventing any unintended padlock ligation or RCA initiation and thereby eliminating time-dependent background growth. Consequently, the cascade can be activated on demand by brief UV exposure, yielding a detection workflow characterized by operational simplicity, ultra-low background, precise temporal control, and enhanced probe stability. By contrast, in a conventional mode with freely mixed gDNA, *pf*Ago, padlock, ligase, and polymerase coexisting throughout incubation, rare nonspecific ligation events and low-level RCA can accumulate over time, leading to progressively elevated background fluorescence. Such baseline drift is a known issue in prolonged isothermal amplifications and necessitates strict gating to maintain assay fidelity.

The logical AND gate is a fundamental building block in digital circuits and, when combined with other elementary operations, enables implementation of arbitrary Boolean functions.^31–33^ In our system, photoactivation serves not merely as a convenient trigger but as an independent biochemical input that, together with target recognition, establishes a strict molecular AND gate governing cascade activation. UV irradiation (Input A) and the target nucleic acid (Input B) function as chemically orthogonal inputs that each regulate a necessary branch of the reaction. Only when both inputs are present (A = 1, B = 1) does the cascade proceed through all checkpoints to generate an output (Fig. 1b). Illumination (A = 1) releases functional gDNA and a ligation-competent padlock, enabling target-dependent RCA and subsequent *pf*Ago activation. By contrast, in the dark (A = 0), the photocleavable precursor retains both the guide and padlock in inactive form, preventing padlock circularization and abolishing RCA initiation as well as *pf*Ago-mediated cleavage (Fig. 1c). In strict accordance with Boolean logic, productive signal turnover occurs exclusively under the dual-input condition; all other input combinations terminate the cascade at defined checkpoints and fail to produce any detectable signal.

The fully enclosed operational workflow is summarized in Fig. 1d. Sample-derived nucleic acids are combined with all reagents, including the photolabile PC-ssDNA encoding the AND gate, in a single tube. The reaction mixture is first subjected to controlled UV irradiation to activate the probe (releasing the gDNA and padlock). This is followed by isothermal incubation at 30 °C to permit padlock ligation and RCA. Finally, a brief heating to 95 °C activates *pf*Ago, which then sequentially processes the RCA products and cleaves the reporter probe to yield a fluorescent readout. This integrated one-pot procedure achieves robust AND gated signal transduction with minimal handling and high operational fidelity.

### Verifying the modular AND-gated cascade design of PANDA

**Fig. 2:**
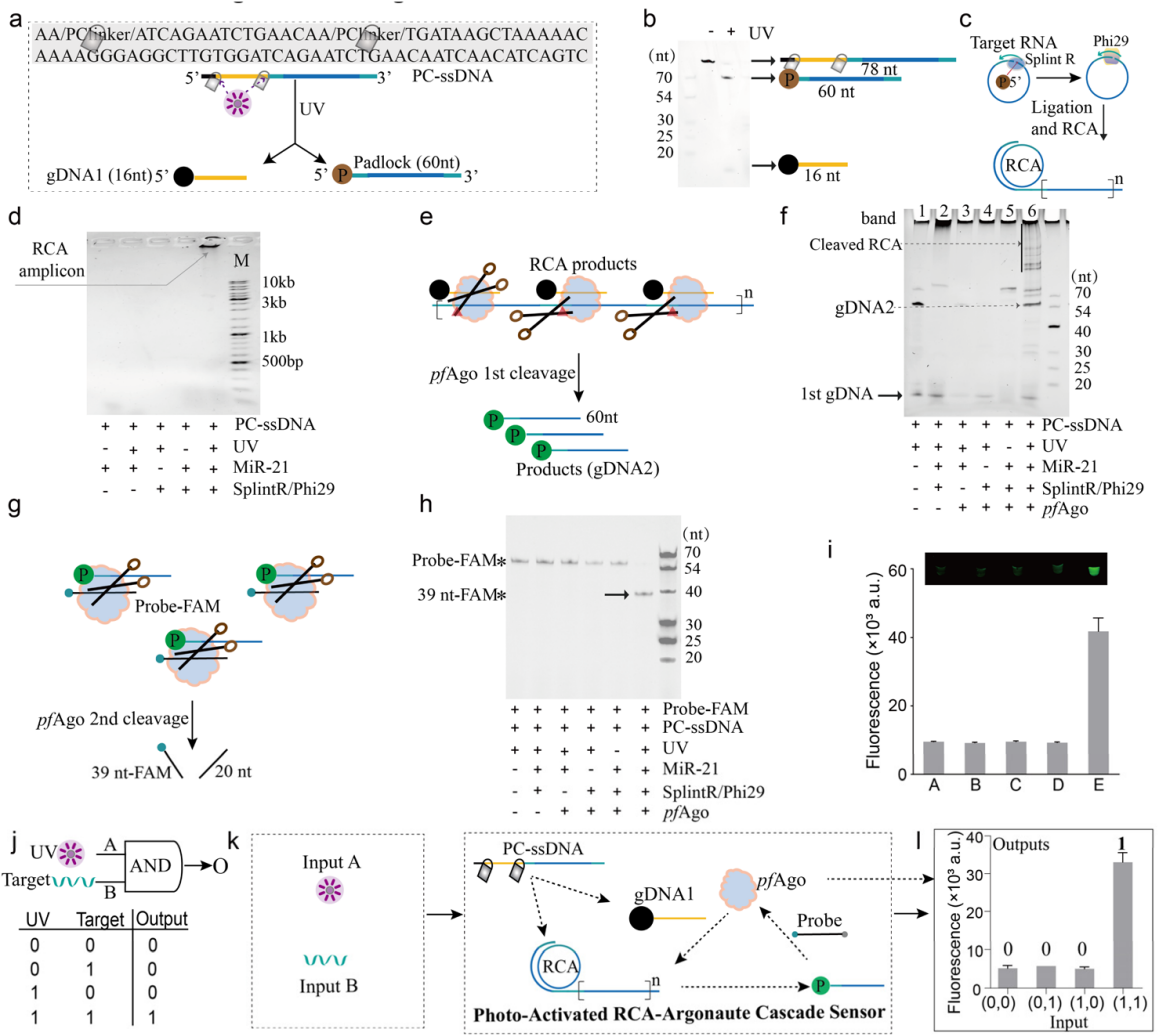
Experimental verification of the PANDA logic-gated reaction framework. (a) Sequence design of the 78-nt PC-ssDNA containing two PC-linkers; upon UV irradiation, generating a 16-nt gDNA1 and a 60-nt padlock probe. (b) Denaturing PAGE analysis of the photocleavage products of PC-ssDNA. (c) Schematic illustration of target-triggered recognition and cyclization of the padlock probe followed by RCA. (d) Agarose gel electrophoresis characterization of RCA product formation. (e) Schematic depiction of *pf*Ago programmed by gDNA1 to recognize and cleave the RCA products. (f) Denaturing PAGE verification of *pf*Ago-gDNA1-mediated cleavage of RCA amplicons. (g) Schematic illustration of secondary probe cleavage by *pf*Ago guided by the newly generated gDNA2. (h) Denaturing PAGE analysis confirming *pf*Ago-gDNA2-mediated cleavage of the FAM-labeled reporter probe. (i) Fluorescence readout demonstrating the overall feasibility of the PANDA cascade, A: probe-FQ only; B: probe-FQ + PC-ssDNA + miR-21 + splintR/phi29 + *pf*Ago; C: probe-FQ + PC-ssDNA + UV + splintR/phi29 + *pf*Ago; D: probe-FQ + PC-ssDNA + UV + miR-21 + *pf*Ago; E: probe-FQ + PC-ssDNA + UV + miR-21 + splintR/phi29 + *pf*Ago. (j) Abstract representation of the AND logic gate, with the corresponding truth table shown below. (k) Mechanistic schematic detailing the implementation of the AND-gated PANDA platform. (l) Experimental fluorescence outputs validating the AND gate behaviour of the PANDA platform.

MiR-21, a microRNA frequently overexpressed in diverse cancers, was selected as a model target to systematically evaluate the performance of the PANDA platform. First, we engineered a 78-nt PC-ssDNA precursor containing two nitrobenzyl PC linkers (Fig. 2a). Upon UV irradiation, both linkers were cleaved in a single activation step, yielding a 16-nt phosphorylated guide DNA (gDNA1) and a 60-nt padlock intermediate. Denaturing polyacrylamide gel electrophoresis (PAGE) analysis validated the efficient photolysis of the precursor into the two expected fragments (Fig. 2b). The released padlock then hybridized with the 21-nt miR-21 target (all sequence details in Fig. S2), allowing splintR ligase to circularize the padlock and initiate RCA (Fig. 2c).

We next tested whether padlock circularization and RCA initiation are jointly gated by both photoactivation and target presence. Agarose gel electrophoresis detected robust high-molecular-weight RCA products only when all required components PC-ssDNA, UV irradiation, and the target RNA were present in the reaction. In contrast, reaction mixtures lacking either input produced no detectable amplification (Fig. 2d), demonstrating that RCA can only be initiated under concurrent UV and target conditions. This built-in dual-input control point ensures that neither input alone yields appreciable background amplification, reinforcing the stringency of the AND-gated design.

We then characterized the downstream processing of RCA amplicons by *pf*Ago (Fig. 2e). In the absence of *pf*Ago, intact high-molecular-weight RCA concatemers remained uncleaved (band 2 in Fig. 2f). Upon addition of *pf*Ago, these long DNA products were efficiently cleaved into a defined set of shorter fragments (band 6 in Fig. 2f), confirming that *pf*Ago can catalytically fragment the RCA amplicons. No cleavage was observed in negative controls lacking either the guide or the upstream RCA step, verifying that *pf*Ago activity in this cascade requires the prior generation of photo-released guide DNA and target-triggered RCA products.

To validate the fidelity of the final signal-generation module, we examined *pf*Ago-mediated cleavage of a fluorescent reporter probe (Fig. 2g). A FAM-labeled single-stranded DNA reporter was designed such that *pf*Ago cutting would produce fragments of defined sizes detectable by denaturing PAGE. Consistent with the upstream results, *pf*Ago generated the expected cleavage bands only in the complete reaction containing UV activation, target RNA, and RCA enzymes (Fig. 2h). An analogous outcome was observed using a fluorophore/quencher (FQ)-labeled reporter probe in a fluorescence readout format (Fig. 2i). In addition, a fluorescence kinetic assay using dual-labeled substrates further supported the stepwise guide-DNA relay, confirming the generation of gDNA2 and its downstream activity (Fig. S3). These results confirm that each layer of the PANDA system-photoactivation, amplification, and Ago-mediated cleavage-operates in a modular yet coordinated manner, yielding a programmable multi-stage reaction pipeline with minimal crosstalk.

Collectively, the above results establish the PANDA cascade as a chemically encoded AND logic gate, wherein UV (Input A) and target RNA (Input B) serve as orthogonal biochemical inputs that must coincide to drive the complete photo-activated RCA-Ago signal generation cascade (Fig. 2j and k). Quantitative fluorescence measurements confirmed a pronounced “ON” state only under the dual-input condition (A = 1, B = 1), whereas all other input combinations remained at the background level (Fig. 2l). This outcome underscores the high fidelity of Boolean gating achieved in a single closed-tube assay, effectively digitizing nucleic acid detection by strictly linking output generation to the AND logic rule. Long-term stability assessment showed that the PC-ssDNA probe remained functionally stable, with no noticeable increase in background fluorescence, even after 7 days of storage at 25 °C under ambient indoor light (Fig. S4). These results underscore the operational robustness of the photolabile probe for practical experimental deployment.

### Optimization and biological validation of the PANDA platform

**Fig. 3:**
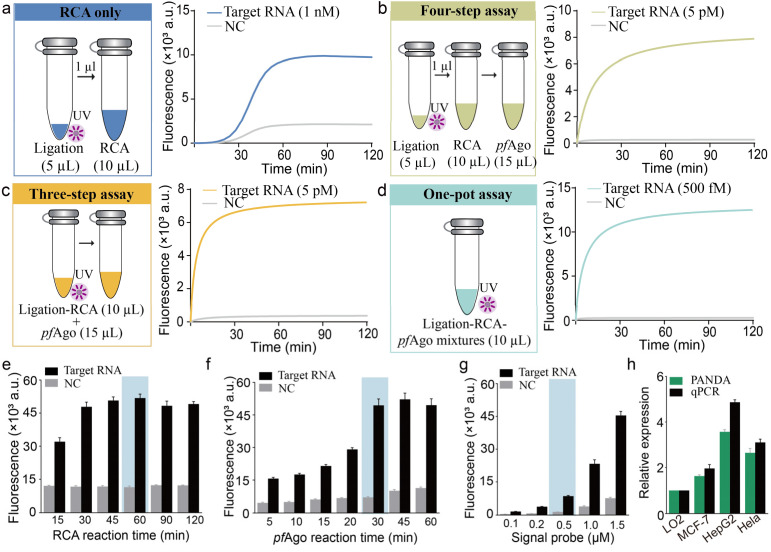
Optimization and the biological validation of the PANDA assay. (a) RCA-only workflow consisting of three sequential steps: UV exposure, padlock probe ligation and RCA. The assay was conducted with 1 nM target RNA. (b) Four-step tandem cascade assay integrating sequential UV exposure, ligation, RCA, and *pf*Ago-induced signal readout. The assay was conducted with 5 pM target RNA. (c) Three-step tandem cascade assay including UV irradiation, ligation-RCA, and *pf*Ago-mediated signal generation. The assay was conducted with 5pM target RNA. (d) One-pot integrated assay in which ligation, RCA, and *pf*Ago-induced readout are conducted in a tube. The assay was conducted with 500 fM target RNA. (e) Time-dependent fluorescence analysis of the RCA reaction from 15 to 120 min. (f) Kinetic fluorescence analysis of *pf*Ago-guided specific cleavage as a function of reaction time. (g) Optimization of signal probe concentration. (h) Quantitative comparison of relative miR-21 expression levels measured by PANDA and RT-qPCR in LO2, MCF-7, HepG2, and HeLa cell lines. Error bars = ± SD; NC = no target control.

To streamline the operational workflow and minimize the risk of aerosol contamination, we developed and evaluated a fully integrated one-pot PANDA assay, in which UV-triggered padlock activation, RCA, and *pf*Ago-mediated DNA cleavage proceed sequentially within a single sealed reaction tube. This configuration was benchmarked against both a conventional multi-step protocol and an RCA-only cascade lacking Ago processing (schematically illustrated in Fig. S5). Real-time fluorescence revealed that the one-pot PANDA assay achieved a detection limit of 500 fM, which is markedly superior to that of the RCA-only method (≈1 nM) and surpasses that of the conventional multi-step workflow (≈5 pM) (Fig. 3a–d). The above results demonstrated that the one-pot format produced substantially higher endpoint fluorescence intensities than the multi-step protocol, reflecting enhanced cascade efficiency under fully enclosed reaction conditions. This performance improvement arises from the elimination of inter-tube transfer steps and the maintenance of optimal local concentrations of RCA products, Ago guides, and reporters, which together reduce material loss and dilution effects while accelerating overall reaction kinetics.

Consequently, the integrated one-pot PANDA strategy delivers a compelling combination of ultrahigh analytical sensitivity, operational simplicity, and assay robustness, providing a practical solution for nucleic acid detection in a sealed, contamination-free format. We next optimized key biochemical parameters by multi-condition fluorescence profiling. 2.5 U µL^−1^ of SplintR ligase and 0.2 U µL^−1^ of phi29 DNA polymerase afforded the most efficient padlock circularization and subsequent RCA, respectively. A PC-ssDNA concentration of 500 nM together with 1 µM *pf*Ago yielded the highest signal-to-background ratio (Fig. S6). Temporal profiling identified a 60 min RCA phase (Fig. 3e), followed by a 30 min *pf*Ago cleavage step (Fig. 3f), in combination with a 500 nM fluorescent reporter (Fig. 3g), as the optimal reaction regime. Under these conditions, the PANDA assay achieved a detection limit of 1.13 fM with excellent linearity (3*σ*/slope, *R*^2^ = 0.9973) and maintained stringent sequence specificity (Fig. S7). To evaluate the performance of this one-pot PANDA assay in biologically relevant samples, we quantified miR-21 expression across a panel of human cell lines, including non-malignant LO2 and three cancer-derived lines (MCF-7, HepG2, and HeLa). The PANDA platform consistently detected elevated miR-21 levels in all cancer cell lines relative to LO2,^34–36^ with expression patterns closely matching RT-qPCR reference measurements (Fig. 3h). These results demonstrate that the optimized PANDA assay enables sensitive and accurate nucleic acid detection in complex cellular matrices, underscoring its potential for practical biological analysis and diagnostic applications.

### Modular multiplexing capability of the PANDA platform

**Fig. 4:**
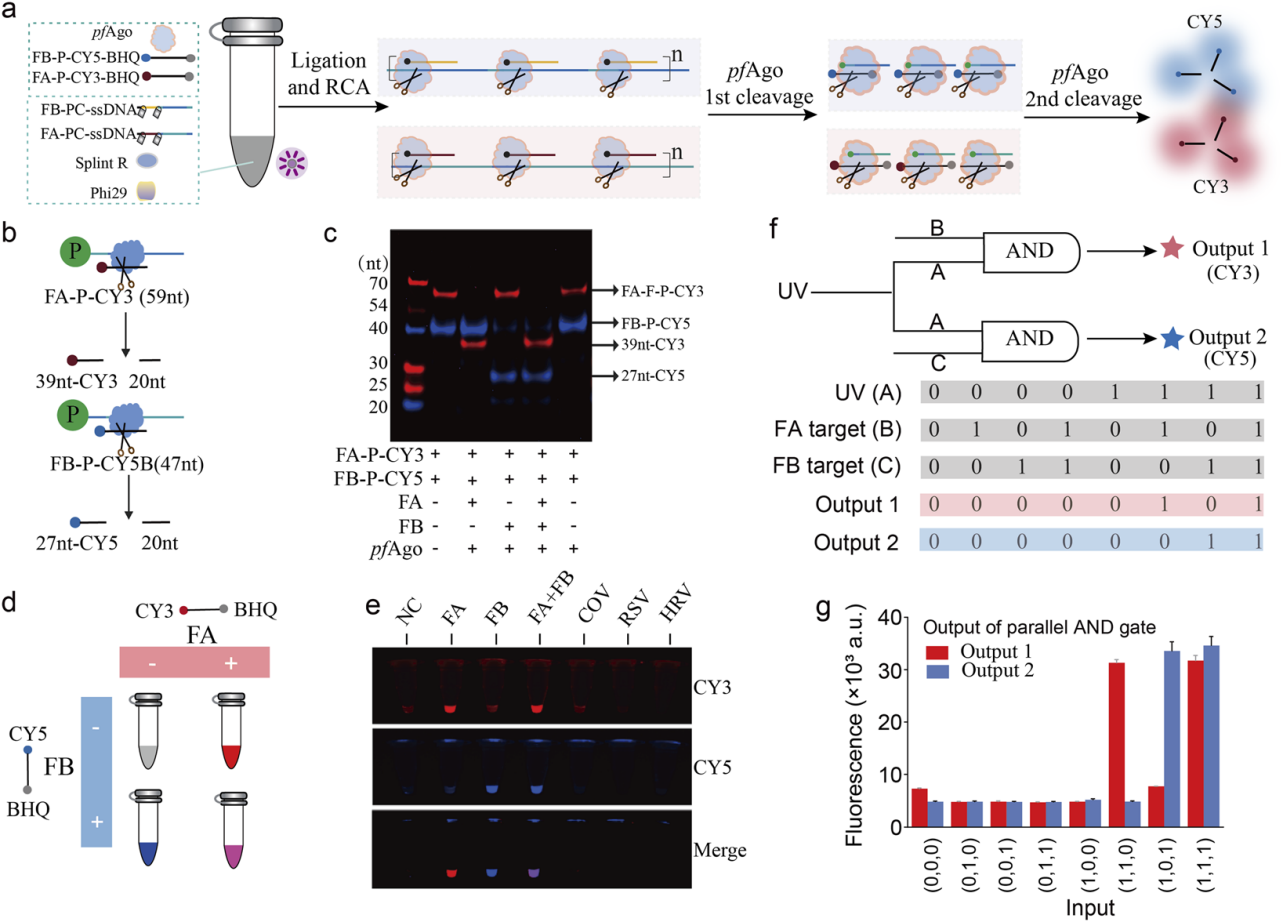
Dual-target detection of FA and FB RNA using the PANDA platform. (a) Schematic workflow of PANDA for the simultaneous detection of FA and FB RNA targets. (b) Design of dual fluorescence reporter probes and their *pf*Ago-mediated cleavage scheme for parallel signal generation. (c) Denaturing PAGE verification of the simultaneous cleavage of both reporter probes, with band positions corresponding to the expected product sizes. (d) Schematic illustration of the fluorescent output patterns for the concurrent detection of FA and FB using orthogonal fluorescence reporters, where FA is recognized by a Cy3/BHQ-labelled probe and FB by a Cy5/BHQ-labelled probe. (e) Photographic images demonstrating the simultaneous detection of FA and FB in the presence of specific targets and related interferents, highlighting the high specificity and orthogonality of the PANDA assay. (f) Abstract representation of the parallel AND logic gate architecture implemented in the dual-channel PANDA system, with the corresponding Boolean truth table shown below. (g) Experimental fluorescence readout validating the parallel AND logic operation, illustrating channel-specific signal activation exclusively under the dual-input conditions.

Simultaneous detection of multiple targets is critical for differential pathogen diagnostics. To endow PANDA with multiplexing capability, we leveraged the modularity of the photocleavable substrates and *pf*Ago reporters to construct orthogonal detection channels. As shown in Fig. 4a, UV activation triggers target-dependent padlock circularization and RCA, providing a unified mechanism for amplification and target discrimination. We selected Influenza A (FA) and Influenza B (FB) viral RNAs as model targets for dual-channel validation (Fig. S8).^37–39^ Two separate PC-ssDNA probes and corresponding fluorophore-labeled reporters were designed for FA and FB, respectively (Fig. 4b). RCA was observed only when both UV and the corresponding target RNA were present (Fig. S9 and S10a), confirming strict dual-input dependence. Native PAGE analysis further showed that *pf*Ago cleaved the reporter probe only in reactions containing the matching target (FA or FB), with no cross-cleavage between channels (Fig. 4c).

We next investigated whether PANDA could enable truly orthogonal dual-channel detection of FA and FB within a single reaction vessel. To this end, we designed two fluorogenic reporter probes with an identical FRET architecture but bearing spectrally distinct fluorophores: a Cy3/BHQ-labelled probe for FA monitoring and a Cy5/BHQ-labelled probe for FB monitoring (Fig. 4d). In their intact states, fluorescence emission from both reporters is efficiently quenched. Upon activation of the corresponding PANDA modules, *pf*Ago catalytically cleaves the reporters, spatially separating the fluorophores from their quenchers and thereby generating channel-specific fluorescence signals that can be directly visualized as distinct color outputs.

We then evaluated assay specificity under multiplex conditions using samples containing FA, FB, or a panel of related interfering targets. As shown in Fig. 4e, S10b and S10c, samples containing FA alone produced a dominant Cy3 fluorescence response, whereas samples containing FB alone generated selective Cy5 signals. In reactions harboring both FA and FB, concurrent activation of both channels yielded a mixed fluorescence output indicative of simultaneous target recognition. In contrast, the presence of noncognate or interfering RNAs failed to induce appreciable fluorescence in either channel. These results demonstrate that PANDA preserves strict channel orthogonality and high sequence specificity during concurrent detection, with negligible cross-activation by off-target inputs. Furthermore, sensitivity testing using serially diluted viral samples established detection limits of 1.27 copies per µL for FA and FB (Fig. S11). Conceptually, the dual-channel configuration can be formalized as a parallel AND logic gate in which each fluorescence output operates as an independent AND gate governed by two orthogonal inputs: a shared optical trigger and a target-specific RNA cue (Fig. 4f). Under this framework, Cy3 emission corresponds to the logical operation AND (UV, FA), while Cy5 emission corresponds to AND (UV, FB). Experimental fluorescence readouts obtained across all combinations of FA and FB inputs (Fig. 4g) conformed precisely to this Boolean behavior: Cy3 signals were observed exclusively under concurrent UV exposure and FA presence, remaining at the baseline under all other conditions, and Cy5 fluorescence was strictly activated only when UV and FB were simultaneously present. Collectively, these findings establish that PANDA implements a parallel AND-gated sensing architecture with robust orthogonal signal routing, thereby enabling simultaneous, interference-free detection of multiple viral targets within a single integrated assay format.

### Clinical validation and expanded applications of the PANDA platform

**Fig. 5:**
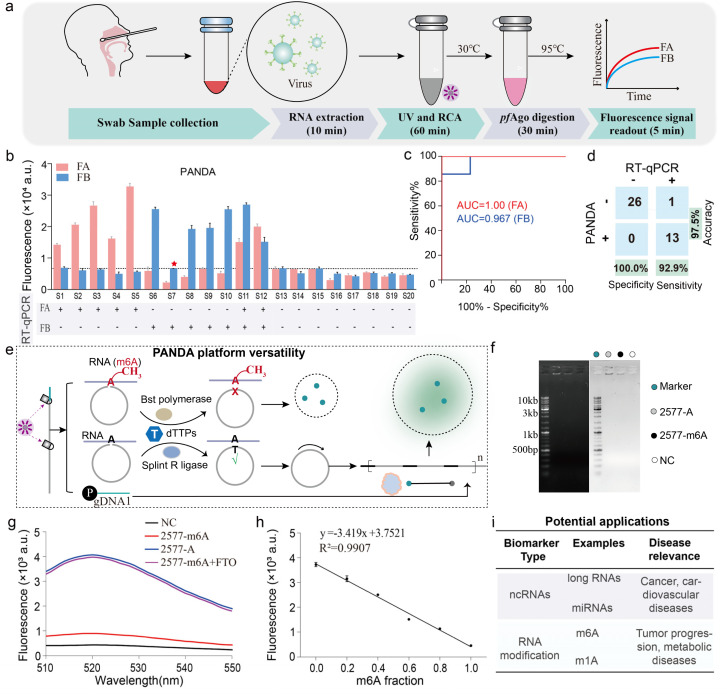
Clinical validation and application prospects of the PANDA platform. (a) Schematic workflow of the PANDA platform for simultaneous detection of FA and FB RNA in clinical samples. (b) Detection outcomes of PANDA obtained from 20 throat swab specimens, with corresponding RT-qPCR results shown below: 5 FA-positive samples, 5 FB-positive samples, 2 co-infection samples (FA/FB), and 8 negative samples. (c) ROC curves generated from the diagnostic results of the 20 clinical specimens for performance evaluation. (d) Confusion matrix comparing PANDA with standard RT-qPCR for clinical diagnosis, yielding a sensitivity of 92.9%, specificity of 100.0%, and overall accuracy of 97.5%. (e) Schematic illustration of the extended application of PANDA for RNA epitranscriptomic analysis, exemplified by detection of m6A-modified RNA sites. (f) Electrophoretic analysis of PANDA for 2577-m6A and 2577-A. (g) Fluorescence analysis of PANDA for 2577-m6A, 2577-A and FTO-treated 2577-m6A. The excitation wavelength was 519 nm. (h) Calibration curve demonstrating the linear relationship between fluorescence intensity and the methylation fraction in standard mixtures of 2577-m6A and 2577-A RNA. (i) Conceptual schematic highlighting the potential future applications of PANDA across diverse nucleic acid biomarker-based disease diagnostics.

We next evaluated the clinical performance of the dual-channel PANDA assay using throat swab specimens. Total RNA was extracted from 20 patient samples and simultaneously tested for FA and FB in a single reaction (Fig. 5a and S12). The PANDA readouts showed near-perfect agreement with standard qRT-PCR results for these samples: out of 20 specimens (5 FA-positive, 5 FB-positive, 2 coinfected with FA + FB, and 8 negatives), only one sample yielded a discrepant result between PANDA and RT-qPCR (Fig. 5b). Receiver operating characteristic (ROC) analysis indicated excellent diagnostic accuracy, with area under the curve values of 1.00 for FA and 0.967 for FB (Fig. 5c). Additionally, a confusion matrix comparison showed that PANDA achieved 92.9% sensitivity, 100% specificity, and 97.5% overall accuracy relative to qRT-PCR (Fig. 5d). Together, these data validate that the PANDA assay can reliably detect viral RNA targets in clinical samples with performance comparable to the gold standard PCR, while providing the advantages of multiplexed one-pot operation.

Beyond dual viral detection, the PANDA architecture provides a general route for extending target scope through padlock-mediated molecular recognition. Because RNA base modifications critically influence ligation efficiency, the platform can be readily adapted for epitranscriptomic profiling.^40,41^ As illustrated in Fig. 5e, *N*^6^-methyl-adenosine (m6A) at the ligation junction sterically impedes padlock circularization, thereby suppressing RCA initiation and subsequent *pf*Ago-mediated cleavage, resulting in complete abrogation of fluorescence output. In contrast, unmethylated RNA enables efficient ligation, cascade activation, and robust signal generation, allowing sensitive discrimination of RNA methylation states using the PANDA workflow. As a proof-of-concept demonstration, we selected a well-characterized m6A site at position 2577 of the metastasis-associated lung adenocarcinoma transcript 1 (MALAT1) long non-coding RNA as a model target. To evaluate the feasibility of the PANDA assay for m6A detection, we first applied it to synthetic MALAT1 RNA oligonucleotides containing either an unmodified adenine (2577-A) or an *N*^6^-methyladenosine (2577-m6A) at this site (sequence listed in Fig. S13). RCA products analyzed by gel electrophoresis revealed substantially greater amplification from the 2577-A (lane 2) compared with the 2577-m6A (lane 3); a control reaction without target RNA showed a nearly undetectable product (lane 4) (Fig. 5f). To directly verify the specificity of the PANDA assay for m6A detection, we treated the 2577-m6A RNA with the m6A demethylase FTO prior to PANDA analysis. FTO-demethylated 2577-m6A produced a fluorescence signal comparable to that of unmodified 2577-A, and substantially higher than that of untreated 2577-m6A, confirming that the signal suppression observed in PANDA is specifically attributable to the m6A modification at the padlock ligation junction (Fig. 5g). Furthermore, a calibration curve relating fluorescence intensity to the methylation fraction was established using predefined mixtures of the 2577-A and 2577-m6A RNA standards (Fig. 5h). Together, these results confirm the programmability of the PANDA assay for detection of site-specific RNA methylation. Furthermore, the modular design of probe and reporter elements enables programmable reconfiguration of the platform for the detection of diverse classes of noncoding RNAs, including miRNAs and long noncoding RNAs. Considering the central regulatory roles of these RNA species in oncogenesis, cardiovascular pathologies, and metabolic disorders, PANDA represents a broadly adaptable molecular sensing platform with strong potential for early disease screening, longitudinal biomarker monitoring, and decentralized diagnostic applications (Fig. 5i). In summary, by integrating optochemical control with catalytic amplification, PANDA offers a unique combination of controllability, multiplexing, and portability that addresses several unmet needs in nucleic acid testing.

## Conclusions

In summary, we have developed PANDA, a photo-activated RCA-Ago cascade that implements a stringent molecular AND logic gate for programmable, multiplexed RNA sensing within a true one-pot format. By integrating optochemical control with catalytic signal amplification, PANDA overcomes key limitations of existing CRISPR-based diagnostics, including nonspecific collateral cleavage, guide instability, and the requirement for physical compartmentalization to enable multiplexing. Compared with existing isothermal amplification-Cas and Ago-based diagnostic platforms (Table S2), PANDA simultaneously achieves true single-tube operation, multiplexing without physical isolation, and stringent signal orthogonality while maintaining high analytical sensitivity.

The modular architecture supports parallel construction of orthogonal logic units synchronized by a common optical trigger, enabling simultaneous detection of multiple RNA targets in a single reaction vessel with minimal operational complexity. Although spectrally distinct fluorescent reporters are used in PANDA, its multiplexing capability arises from the underlying reaction mechanism rather than the reporter format itself. Specifically, strictly guide-dependent Ago cleavage, together with dual-input gating, enables orthogonal multi-channel detection in a shared reaction environment without physical separation, while minimizing the cross-reactivity often associated with collateral cleavage in CRISPR-based systems. Beyond analytical performance, we demonstrate the clinical applicability of PANDA using real patient samples, achieving diagnostic accuracy comparable to RT-qPCR while offering superior workflow simplicity and multiplexing capability. Moreover, the generality of the padlock ligation strategy extends the platform beyond viral RNA detection to epitranscriptomic profiling and non-coding RNA analysis, establishing PANDA as a versatile framework for biomarker interrogation. Collectively, this work introduces a generalizable strategy for constructing logic-gated biomolecular circuits that enable coordinated signal processing in complex sensing environments, bridging molecular computation and practical diagnostics. PANDA thus represents a significant step toward stable, field-deployable, and digitally programmable nucleic acid testing platforms.

## Ethical statement

All experiments were performed in accordance with the Declaration of Helsinki guidelines, and approved by the ethics committee at Zhejiang University. Informed consent was obtained from the human participants of this study.

## Author contributions

T. H. applied for the funding. T. H., C. X. C., and C. Y. H. conceived the idea and supervised the project. X. X. K., J. W. Q., and J. L. W. performed the experiments and data collection. X. X. K., T. F. X., Y. L. W., and T. H. analyzed the data. X. X. K., H. M. F., and T. H. wrote the manuscript.

## Conflicts of interest

There are no conflicts to declare.

## Supplementary Material

SC-OLF-D6SC00827E-s001

## Data Availability

The data supporting this article have been included as part of the supplementary information (SI). Supplementary information: experimental section, sequences of oligonucleotides, comparison with one-pot RNA detection and demographic information involved in the experiments. Supplementary information is available. See DOI: https://doi.org/10.1039/d6sc00827e.
