# Supplementary material for: PANDA: AND logic-gated RNA sensing enabled by a photo-activated RCA-Argonaute cascade
Source: Chem Sci. 2026 Apr 9;17(21):10643–52. doi: 10.1039/d6sc00827e (PMC13090041; doi:10.1039/d6sc00827e)
Supplement: SC-017-D6SC00827E-s001 [file SC-017-D6SC00827E-s001.pdf]

Supplementary Information (SI) for Chemical Science.
This journal is © The Royal Society of Chemistry 2026

Supplementary Materials

PANDA: AND Logic-Gated RNA Sensing Enabled by Photo-Activated RCA-Argonaute Cascade

EXPERIMENTAL SECTION

Clinical Samples and Ethics Statement. Clinical throat swab samples (n = 20) used in this study were collected from the Children's Hospital of Zhejiang University School of Medicine, which had been approved by the Ethical Committee of the Children's Hospital of Zhejiang University School of Medicine (2025-IRB-0420-P-01). All samples were preserved in virus-inactivating preservation solution immediately after collection to ensure biosafety and prevent sample degradation. Sample handling strictly followed standard operating procedures for viral nucleic acid detection, including the use of sterile sampling tools and aseptic techniques to avoid cross-contamination.

Materials and Apparatus. All oligonucleotides used in the experiment were synthesized by General Biol (Anhui) Co., Ltd. (Chuzhou, China). The DNA sequences are listed in Table S1. Flu A and Flu B DNA gene fragments with a T7 promoter were in vitro transcribed using the T7 High Yield RNA Transcription Kit to generate synthetic RNA targets. The RaPure Viral RNA/DNA Kit, miRNA 1st Strand cDNA Synthesis Kit (by stem-loop), and miRNA Universal SYBR qPCR Master Mix were obtained from Vazyme (Nanjing, China). The TransScript Uni All-in-One First-Strand cDNA Synthesis SuperMix was purchased from Transgen Biotech (Beijing, China), and the TB Green Fast qPCR Mix was acquired from Takara (Tokyo, Japan). FTO (mg mL^{-1}), Phi29 polymerase (10 U μL^{-1}), dNTPs (2.5 mM), SplintR ligase (25 U μL^{-1}), T4 DNA ligase (1000 U μL^{-1}), T7 High Yield RNA Transcription Kit, PAGE-related reagents [acryl/bis 30% solution (29:1), Gel-Red], and His-tag Protein Purification Kit were provided by Beyotime Biotechnology (Shanghai, China). Bst 2.0 warm startDNA polymerase was purchased by New England Biolabs (Beijing, China).

The CFX Opus 96 Real-Time PCR System was used for qPCR analysis. A chemiluminescence imaging system (Bio-Rad Laboratories, Inc., Hercules, CA, USA) was employed for gel scanning and tube imaging. Fluorescence data were acquired using a microplate reader (Tecan Spark GmbH, Austria).

Cell Culture and Total RNA Extraction. The human normal liver cell line LO2, human hepatocellular carcinoma cell line HepG2, human cervical carcinoma cell line HeLa, and human breast adenocarcinoma cell line MCF-7 were cultured in high-glucose Dulbecco's Modified Eagle Medium (DMEM, Gibco, USA) supplemented with 10% fetal bovine serum (FBS, Thermo Fisher Scientific, USA) and 1% penicillin-streptomycin (100 U/mL penicillin and 100 μg/mL streptomycin, Gibco, USA) under a humidified atmosphere containing 5% CO_2 at 37 °C.

For total RNA extraction, cells were harvested and resuspended in 1 mL TRIzol® Reagent (Invitrogen, USA) per 1×10^6 cells, and total RNA was extracted according to the manufacturer's manual. The extracted RNA was stored at -80 °C to prevent degradation.

Gel Electrophoretic Analysis. Polyacrylamide Gel Electrophoresis (PAGE) Analysis: Nucleic acids were heated at 95°C for five minutes before loading onto a 20% denaturing PAGE gel. After 90 minutes of electrophoresis at 120 V, the gels were stained with or without Gel-Red for scanning.

Agarose Gel Electrophoresis: Gels containing Gel-Red were prepared using 0.5× TBE buffer (which also served as the electrophoresis buffer). The gels were run at 140 V for 30 minutes.

Expression and Purification of *Pf*Ago. The constructed recombinant plasmid for the expression of N-terminal His-tagged *pf*Ago was transformed into Escherichia coli BL21(DE3) cells. The transformants were inoculated in 20 mL LB broth (supplemented with ampicillin, 100 μg/mL) and incubated overnight. The overnight cultures were then added to 2 L LB broth and grown at 37 °C until the OD_{600} reached 0.6. Isopropyl β-D-1-thiogalactopyranoside (IPTG) was added to a final concentration of 1 mM to induce protein expression at 16°C for 16 h. The cells were collected by centrifugation (8000 rpm, 5 min at 4°C), resuspended at 4°C in lysis buffer (20 mM Tris/HCl pH 8.0, 500 mM NaCl), and disrupted using an Ultrasonic Homogenizer. The cell lysate was heated at 80°C for 30 min and centrifuged (12000 rpm, 30 min at 4°C). The supernatant containing *pf*Ago was collected and purified using a His-tag Protein Purification Kit. A 50 kDa ultrafiltration column (Millipore, Darmstadt, Germany) was used to concentrate the *pf*Ago protein. The purified protein was stored at -80 °C.

Procedure of the One-Pot Assay. For one-pot operation, all reaction components were premixed directly in the bottom of a single reaction tube. *Pf*Ago (1 μL), 10× reaction buffer (1 μL; 100 mM HEPES, pH 7.5, 500 mM NaCl, 100 mM $MnCl_2$), and the fluorogenic reporter probe (1 μL, 0.5 μM) were first combined with the ligation-RCA reaction mixture to ensure complete buffer compatibility across all enzymatic modules. The ligation and RCA system (total volume, 7 μL) consisted of PC-ssDNA (1 μL), RNA target (1 μL), dNTPs (0.7 μL), phi29 DNA polymerase (0.2 μL), SplintR ligase (0.2 μL), 10× reaction buffer (1 μL), and nuclease-free water to adjust the final volume. All components were gently mixed to homogeneity prior to UV activation and subsequent isothermal incubation. Reagent concentrations were adjusted as required in optimization experiments. The assay was initiated by exposure to UV (365 nm, 40 W, 10 s), and the tube was incubated at 30 °C for a specific period. After incubation, the mixture was heated to 95 °C to activate the *pf*Ago reaction. The assay was performed in a qPCR device at 95 °C for a specific period, with fluorescence intensity monitored every 2 minutes. The final fluorescence intensity was recorded using a microplate reader.

Procedures of RCA-Only, Four-Step, and Three-Step Methods. RCA-Only Method: A mixture of PC-ssDNA (1 μL) and RNA (1 μL) was first exposed to UV for 10 s, then incubated at 90 °C for 1 min and gradually cooled to room temperature. Subsequently, 0.5 μL of SplintR buffer, 0.25 μL of SplintR ligase, and 2.25 μL of nuclease-free water were added to the mixture. Ligation was performed at 25 °C for 1 h, followed by inactivation at 65 °C for 20 min. Then, 1 μL of the ligation product was mixed with a 9 μL RCA reaction system containing 0.2 μL of Phi29 polymerase, 0.7 μL of dNTPs, 1 μL of 10× SYBR Green II, 1 μL of Phi29 buffer, and 6.1 μL of nuclease-free water. RCA was performed at 30 °C for 2 h in a qPCR device, with monitoring every 2 min.

Four-Step Method: The ligation and RCA steps were the same as those in the RCA-only method, followed by enzyme inactivation at 65 °C for 10 min. A mixture containing 1 μL of *pf*Ago, 1 μL of signal probe (0.5 μM), 1.5 μL of 10× buffer (100 mM HEPES pH 7.5, 500 mM NaCl, 100 mM $MnCl_2$), and 1.5 μL of nuclease-free water was added to the RCA reaction system, and the assay was performed at 95 °C for 2 h in a qPCR device.

Three-Step Method: 1 μL of PC-ssDNA, 1 μL of RNA, 0.7 μL of dNTPs, 0.2 μL of Phi29 polymerase, SplintR ligase, 1 μL of 10× buffer, and 6.1 μL of nuclease-free water were mixed and exposed to UV for 10 s. Then, ligation and RCA were carried out at 30 °C for 2 h. Afterward, the mixture was heated to 95 °C, followed by the addition of 1 μL of *pf*Ago, 1.5 μL of 10× buffer (100 mM HEPES pH 7.5, 500 mM NaCl, 100 mM $MnCl_2$), 1 μL of signal probe (0.5 μM), and 1.5 μL of nuclease-free water. The final reaction was performed at 95 °C for 2 h in a qPCR device.

Procedures of PANDA for m6A Site Detection. *Pf*Ago (1 μL), 10× reaction buffer (1 μL; 100 mM HEPES, pH 7.5, 500 mM NaCl, 100 mM $MnCl_2$), and the fluorogenic reporter probe (1 μL, 0.5 μM) were combined with the ligation-RCA reaction. The ligation and RCA system (total volume, 7 μL) consisted of PC-ssDNA (1 μL), RNA target (1 μL), dNTPs (0.7 μL), 1 μL of Bst 2.0 warm startDNA polymerase (0.4 U/μL), phi29 DNA polymerase (0.2 μL), BSA (0.2 μL), SplintR ligase (0.2 μL), 10× reaction buffer (1 μL), and nuclease-free water to adjust the final volume. All components were gently mixed to homogeneity prior to UV activation and subsequent isothermal incubation. Reagent concentrations were adjusted as required in optimization experiments. The assay was initiated by exposure to UV (365 nm, 40 W, 10 s), and the tube was incubated at 30 °C for a specific period. After incubation, the mixture was heated to 95 °C to activate the *pf*Ago reaction. For m6A demethylase FTO treatment, the 2577-m6A RNA was treated with 1 μg FTO in demethylation buffer (50 mM Tris-HCl, pH 7.5, 300 μM $(NH_4)_2Fe(SO_4)_2$, 300 μM α-ketoglutarate, 2 mM L-ascorbic acid) at 37°C for 2 hours. After heat inactivation of FTO at 65°C for 5 min, the demethylated product was directly applied to the PANDA assay alongside the untreated 2577-m6A and unmodified 2577-A controls. The final fluorescence intensity was recorded using a microplate reader.

qPCR Detection of miRNAs. cDNA was synthesized via the stem-loop method using the miRNA 1st Strand cDNA Synthesis Kit according to the manufacturer's instructions. qPCR was performed with the miRNA Universal SYBR qPCR Master Mix Kit. The 20 μL reaction system contained 2× miRNA Universal SYBR qPCR Master Mix, universal reverse Q primer, specific forward primer, and cDNA template. Each sample included 3 technical replicates, along with blank controls (no cDNA template) and negative controls. The qPCR program was as follows: 95°C for 30 s, followed by 40 cycles of 95°C for 10 s and 60°C for 30 s, and a final melting curve analysis. Ct values were analyzed using the $2^{\wedge(-\Delta\Delta Ct)}$ method.

qPCR Detection of Viral RNA. Viral RNA samples were extracted using the RaPure Viral RNA/DNA Kit and converted into cDNA via the TransScript Uni All-in-One First-Strand cDNA Synthesis SuperMix. qPCR was conducted with specific primers and TB Green Fast qPCR Mix according to the manufacturer's instructions.

Statistical analysis. Descriptive statistics (mean, standard deviation [SD], standard error [SE]) were calculated utilizing standard computational formulas in Microsoft Excel 365. For pairwise comparisons between two independent groups, a two-tailed unpaired Student's T-test was applied. P-values less than 0.05 were considered significant. Receiver operating characteristic (ROC) curve analyses were conducted to evaluate the diagnostic performance, and the area under the ROC curve (AUC) values were generated using GraphPad Prism version 10.0 (GraphPad Software Inc., San Diego, CA, USA).

Supplementary figures

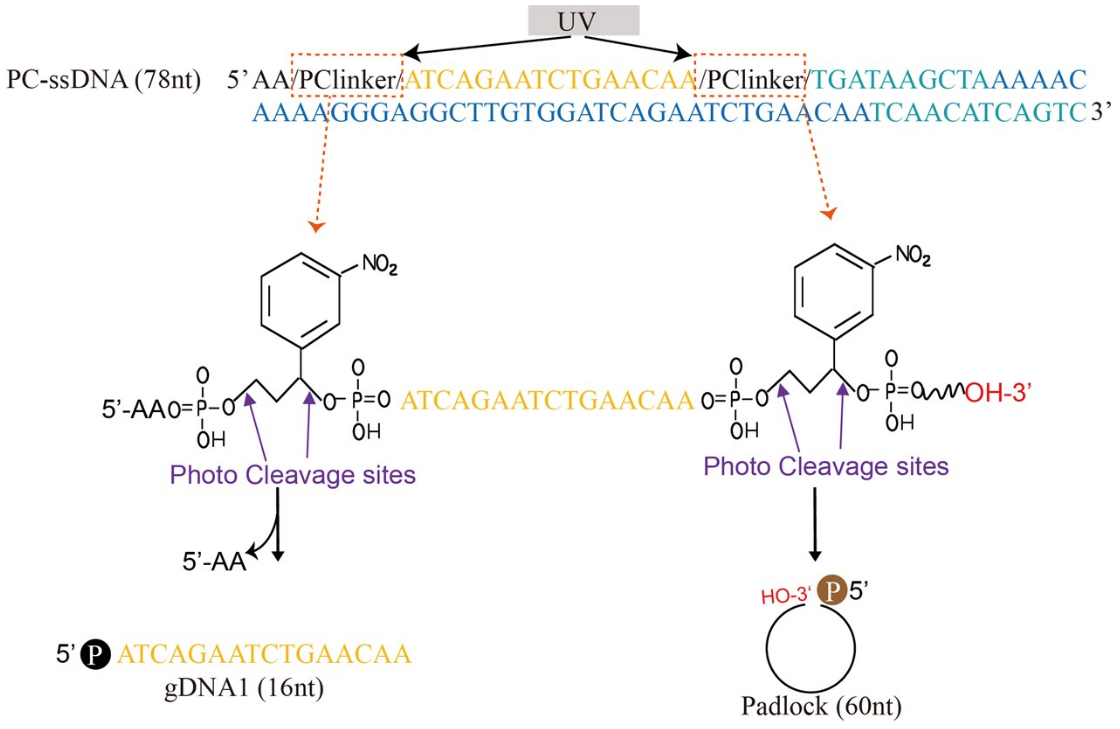

Fig. S1 Structural schematic of the PC-ssDNA probe incorporating two photocleavable (PC) linkers.

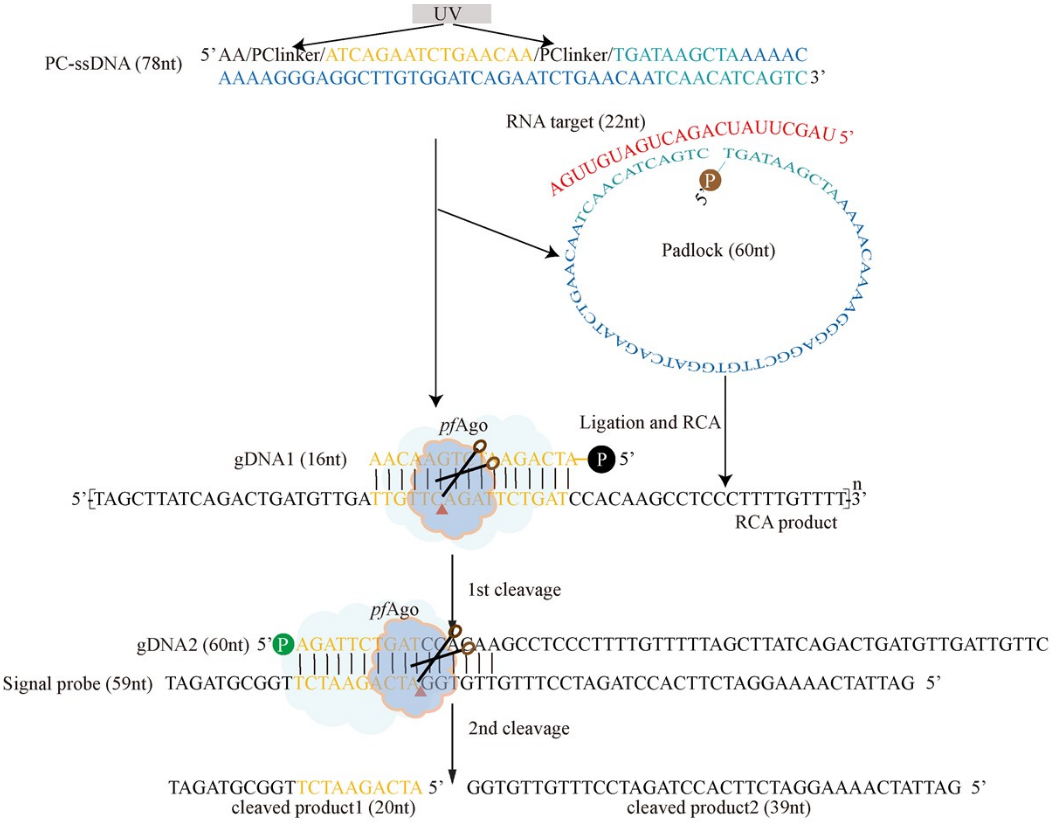

Fig. S2 Detailed sequence choreography of the UV-triggered RCA-pfAgo cascade for recognition of miR-21 target.

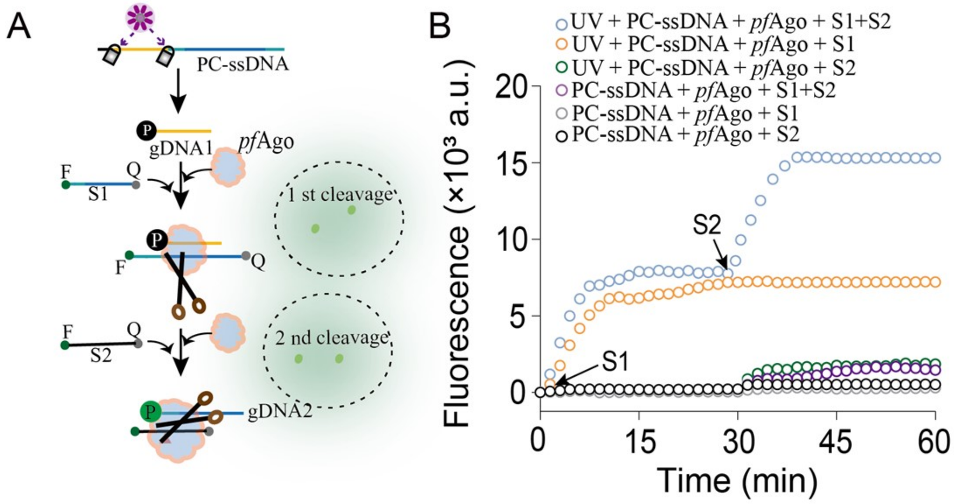

Fig. S3 Kinetic analysis of *pf*Ago-mediated sequential cleavage behaviour. Reactions were performed at 95 °C. S1 (FAM/BHQ1-labeled gDNA1 substrate, 500 nM) was added at time 0, and S2 (FAM/BHQ1-labeled gDNA2 substrate, 500 nM) was introduced at 30 min. Curves correspond to: UV-irradiated PC-ssDNA + *pf*Ago + S1 + S2 (blue); UV-irradiated PC-ssDNA + S1 (yellow); UV-irradiated PC-ssDNA + *pf*Ago + S2 (green); PC-ssDNA + *pf*Ago + S1 + S2 (purple); PC-ssDNA + *pf*Ago + S1 (gray); PC-ssDNA + *pf*Ago + S2 (black). Fluorescence intensity was recorded at 1.5-min intervals.

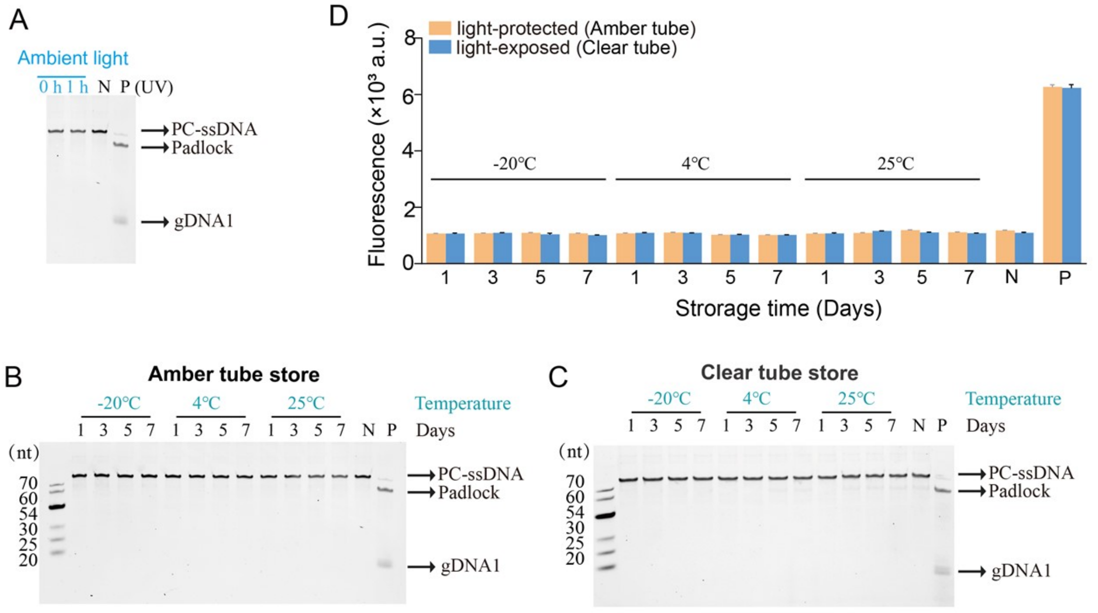

Fig. S4 Long-term stability and ambient light resistance of the PC-ssDNA probe. (A) Denaturing PAGE analysis of PC-ssDNA probe integrity after 1 hour under ambient light. (B) Denaturing PAGE analysis of PC-ssDNA probe integrity after storage at -20℃, 4℃, and 25℃ for 1, 3, 5, and 7 days with the use of amber tube. (C) Denaturing PAGE analysis of PC-ssDNA probe integrity after storage at -20℃, 4℃, and 25℃ for 1, 3, 5, and 7 days with the use of clear tube. (D) Fluorescence-based evaluation of PANDA assay performance using 100 fM miR-21 as the target, comparing light-protected and light-exposed probe storage conditions across the same storage temperatures and durations as in (B) and (C). N: negative control; P: positive control (30s UV-irradiated probe).

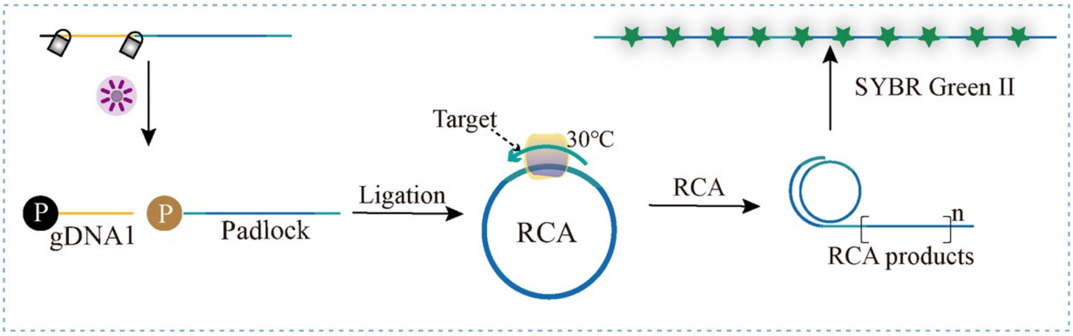

Fig. S5 A schematic illustration of the RCA only assay.

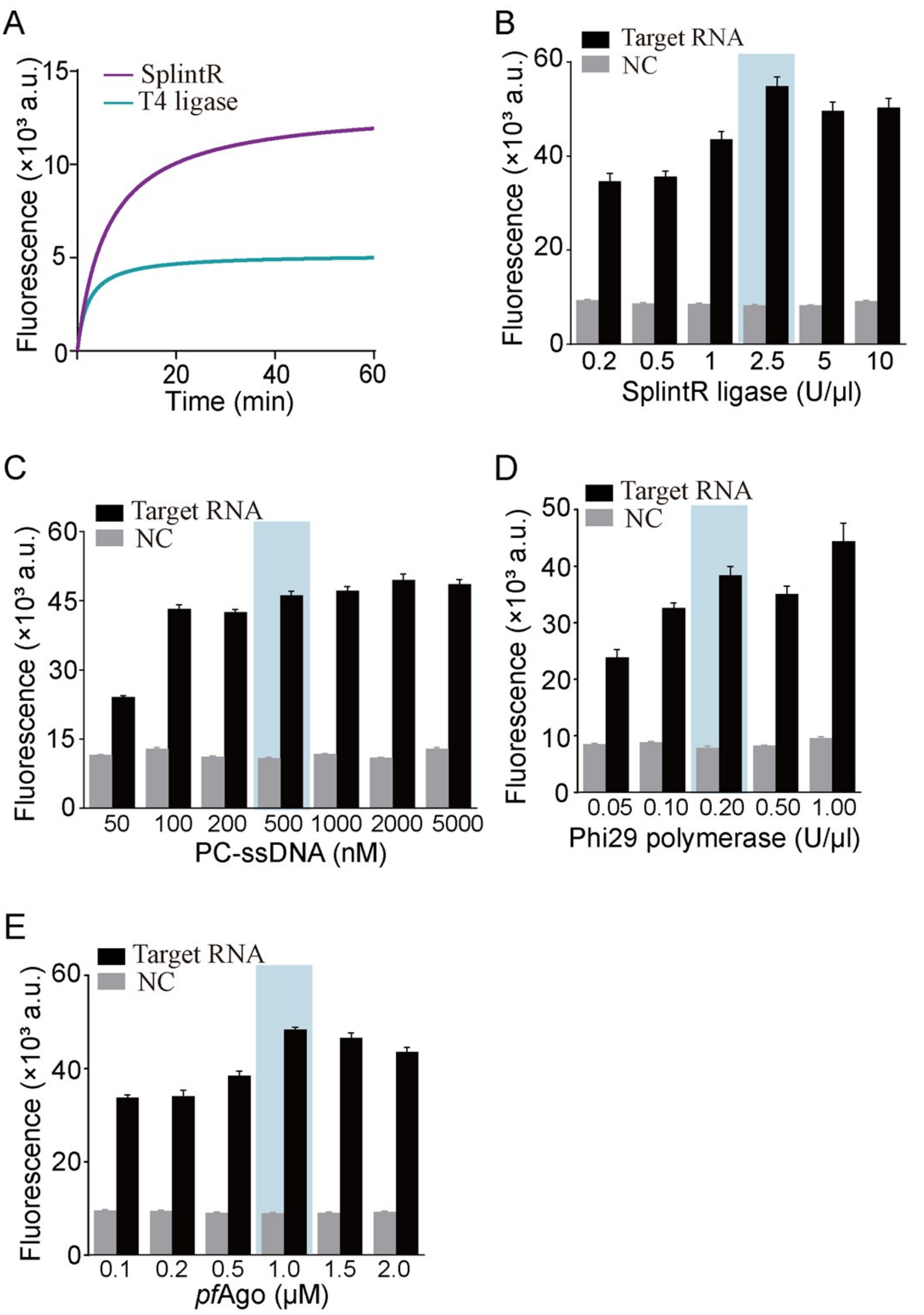

Fig. S6 Fluorescence-based optimization of the PANDA platform. (A) Comparison of T4 DNA ligase. (B) The Splint R concentration of 0.2, 0.5, 1, 2.5, 5 and 10 U/mL. (C) The PC-ssDNA concentration of 50, 100, 200, 500, 1000, 2000, 5000 nM. (D) The phi29 polymerase concentration of 0.05, 0.1, 0.2, 0.5 and 1 U/μL. (E) The *pf*Ago concentration of 0.1, 0.2, 0.5, 1.0, 1.5, 2.0 μM.

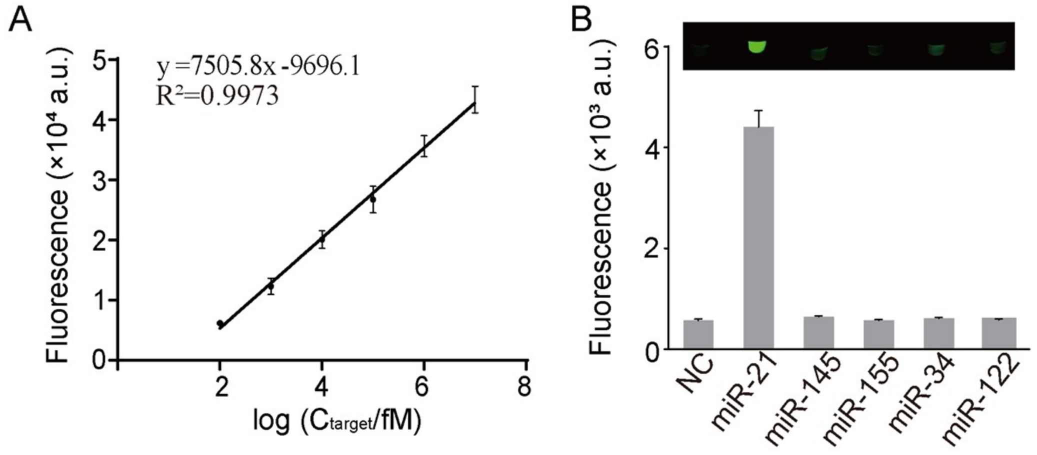

Fig. S7 The performance of the PANDA platform. (A) Linear calibration obtained with the optimal assay conditions. (B) Specificity of the assay for detecting miR-21 against different miRNAs. Error bars represent mean ± SD. for 3 technical replicates.

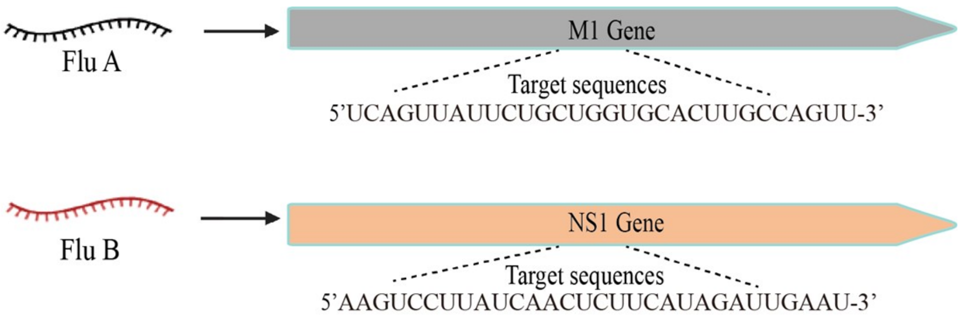

Fig. S8 Schematic diagrams of the full-length M1 gene of FA and NS1 gene of FB with target sites.

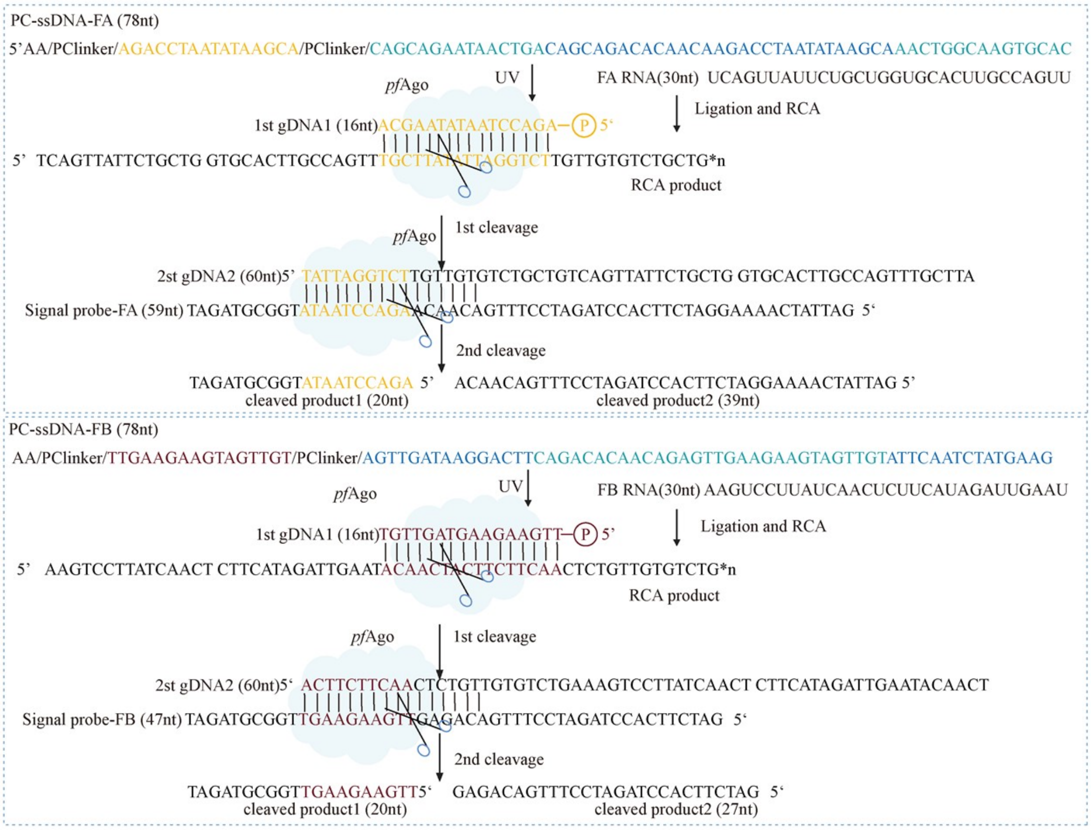

Fig. S9 Detailed sequence choreography of the UV-triggered RCA-Ago cascade for FA and FB detection.

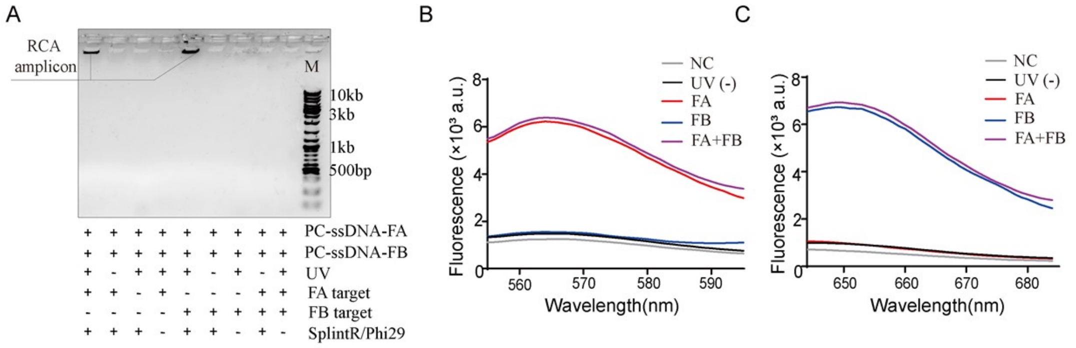

Fig. S10 The feasibility verification of the dual-detection platform. (A) Agrose gel analysis of the ligation and RCA products. (B) Fluorescence excitation spectrum for CY3 signal. C. Fluorescence excitation spectrum for CY5 signal.

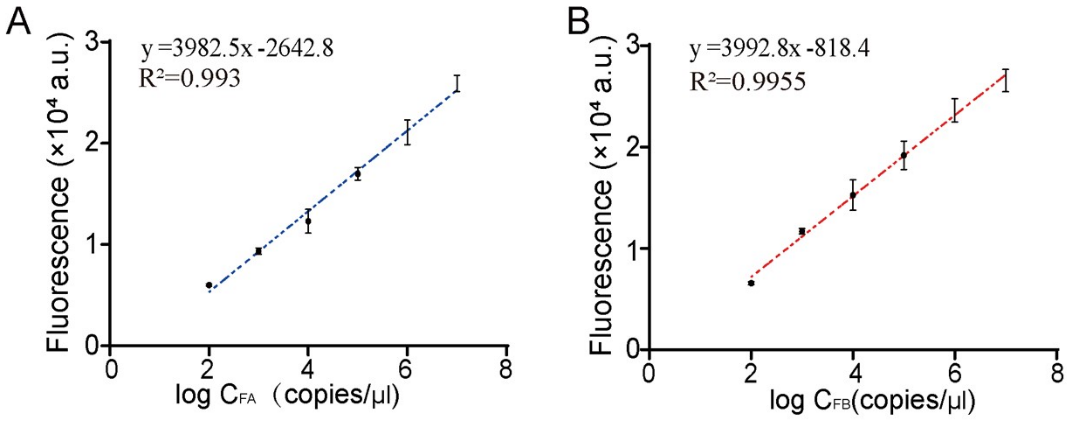

Fig. S11 The sensitivity of the dual-detection platform. (A) Linear calibration for FA detection. (B) Linear calibration for FB detection.

Information of Influenza virus samples

No	Age	Symptoms
1	5 years	FA
2	1 years	FA
3	3 years	FA
4	8 years	FA
5	7 years	FA
6	3 years	FB
7	< 1 years	FB
8	2 years	FB
9	5 years	FB
10	2 years	FB
11	/	FA/FB
12	/	FA/FB
13	3 years	Normal
14	2 years	Normal
15	7 years	Normal
16	8 years	Normal
17	4 years	Normal
18	3 years	Normal
19	9 years	Normal
20	4 years	Normal

Fig. S12 Summary of clinical information for 20 influenza virus samples. Samples No. 11 and No. 12 were prepared as mixed specimens containing both FA and FB RNA.

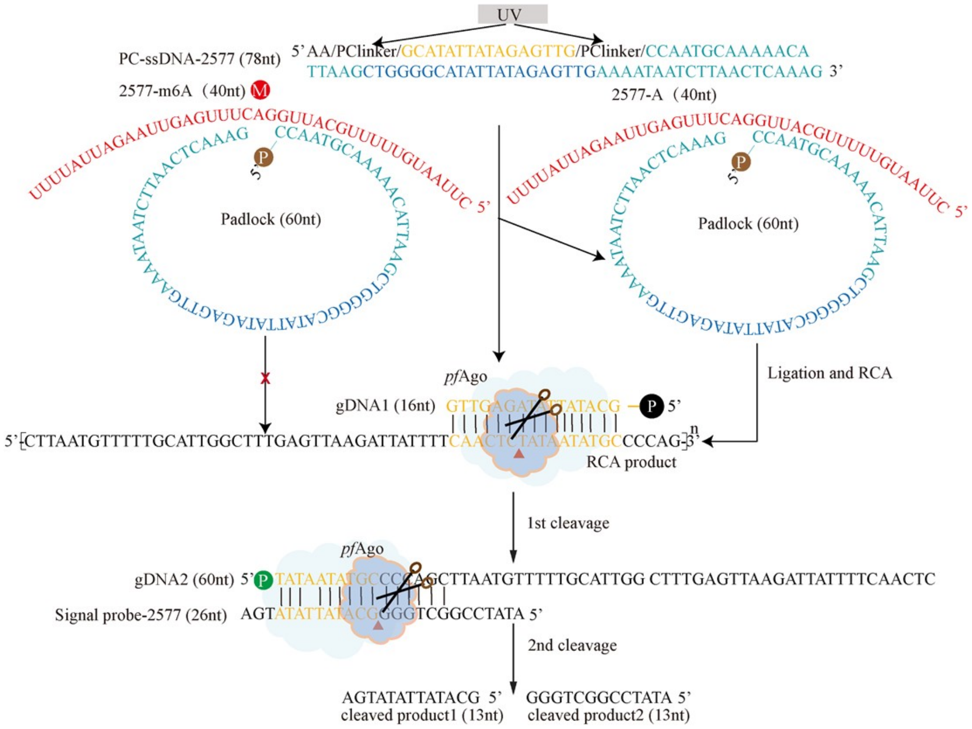

Fig. S13 Detailed sequence choreography of the UV-triggered RCA-Ago cascade for MALAT1 2577 m6A methylation detection.

Table S1. Oligonucleotides used for testing in this study.

Name	Sequence (5'-3')
PC-ssDNA	AA/PClinker/ATCAGAATCTGAACAA/PClinker/TGATAAGCTAAAAACAAAAGGGAGG CTTGTGGATCAGAATCTGAACAATCAACATCAGTC
Signal probe	GATTATCAAAAGGATCTTCACCTAGATCCTTTGTTGTGGATCAGAATCTTGGCGTA GAT
S1	TAGCTTATCAGACTGATGTTGATTGTTCAGATTCTGATCCACAAGCCTCCCTTTTGT TTT
PC-ssDNA-FA	AA/PClinker/AGACCTAATATAAGCA/PClinker/CAGCAGAATAACTGACAGCAGACAC AACAAGACCTAATATAAGCAAACTGGCAAGTGCAC
Signal Probe-FA	GATTATCAAAAGGATCTTCACCTAGATCCTTTGACAACAAGACCTAATATGGCGTA GAT
PC-ssDNA-FB	AA/PClinker/TTGAAGAAGTAGTTGT/PClinker/AGTTGATAAGGACTTCAGACACAAC AGAGTTGAAGAAGTAGTTGTATTCAATCTATGAAG
Signal Probe-FB	GATCTTCACCTAGATCCTTTGACAGAGTTGAAGAAGTTGGCGTAGAT
PC-ssDNA-2577	AA/PCiInker/GCATATTATAGAGTTG/PCiInker/CCAATGCAAAAACATTAAGCTGGGG CATATTATAGAGTTGAAAATAATCTTAACTCAAAG
Signal Probe-2777	ATATCCGGCTGGGGCATATTATATGA
MiR-21	UAGCUUAUCAGACUGAUGUUGA
MiR-145	GUCCAGUUUUCCCAGGAAUCCCU
MiR-155	UUAAUGCUAAUCGUGAUAGGGGUU
MiR-34	UGGCAGUGUCUUAGCUGGUUGUU
MiR-122	UGGAGUGUGACAAUGGUGUUUG
Flu A RNA target	GAAATTAATACGACTCACTATAGGGCTCTCATGGAATGGCTAAAGACAAGACCAA TTCTGTCACCTCTGACTAAGGGGATTTTGGGGTTTGTGTTCACGCTCACCGTGCCCA GTGAGCGAGGACTGCAGCGTAGACGCTTTGTCCAAAATGCCCTCAATGGGAATGG AGACCCAAATAACATGGACAAAGCAGTTAAACTGTATAGGAAACTTAAGAGGGAG ATAACGTTCCATGGGGCCAAAGAAATAGCTCTCAGTTATTCTGCTGGTGCACTTGC CAGTTGCATGGGCCTCATATACAATAGGATGGGGGCTGTAACCACTGAAGTGGCAT TTGGCCTGGTGTGTGCAACATGTGAGCAGATTGCTGACTCCCAGCACAGGTCTCAT AGGCAGATGGTGGCAACAACCAATCCATTAATAAAACATGAGAACAGAATGGTTT TGGCCAGCACTACAGCTAAGGCTATGGAGCAAATGGCTGGATCAAGTGAGCAGGC AGCGGAGGCCATGGAGATTGCTAGTCAGGCCAGGCAGATGGTGCAGGCAATGAGA GCCATTGGGACTCATCCTAGTTCCAGTACTGGTCTAAGGGATGATCTTCTTGAAAA TTTGCAGACCT
Flu B RNA target	GAAATTAATACGACTCACTATAGTAAAGGATGAAGTAAACACTCAGAAAGAGGGG AAATTCCGTTTGACAATAAAAAGGGATATACGTAATGTGTTGTCCTTGAGAGTGTT GGTGAACGGAACCTTCCTCAAGCACCCTAATGGAGACAAGTCCTTATCAACTCTTC ATAGATTGAATGCATATGACCAGAATGGAGGGCTTGTTGCTAAACTTGTTGCTACT GATGATCTTACAGTGGAGGATGAAAAAGATGGCCATCGGATCCTCAACTCACTCTT CGAGCGTTTTGATGAAGGACATTCAAAGCCAATTCGAGCAGCTGAAACTGCGGTG GGAGTCTTATCCCAATTTGGTCAAGAGCACCGATTATCACCAGA

COV<br>RNA target	GAAATTAATACGACTCACTATAGATGTCTGATAATGGACCCCAAAATCAGCGAAAT<br>GCACCCCGCATTACGTTTGGTGGACCCTCAGATTCAACTGGCAGTAACCAGAATGG<br>AGAACGCAGTGGGGCGCGATCAAAACAACGTCGGCCCCAAGGTTTACCCAATAAT<br>ACTGCGTCTTGGTTCACCGCTCTCACTCAACATGGCAAGGAAGACCTTAAATTCCC<br>TCGAGGACAAGGCGTTCCAATTAACACCAATAGCAGTCCAGATGACCAAATTGGC<br>TACTACCGAAGAGCTACCAGACGAATTCGTGGTGGTGACGGTAAAATGAAAGATC<br>TCAGTCCAAGATGGTATTTCTACTACCTAGGAACTGGGCCAGAAGCTGGACTTCCC<br>TATGGTGCTAACAAAGACGGCATCATATGGGTTGCAACTGAGGGAGCCTTGAATA<br>CACCAAAAGATCACATTGGCACCCGCAATCCTGCTAACAATGCTGCAATCGTGCTA<br>CAACTTCCTCAAGGAACAACATTGCCAAAAGGCTTCTACGCAGAAGGGAGCAGAG<br>GCGGCAGTCAAGCCTCTTCTCGTTCCTCATCACGTAGTCGCAACAGTTCAAGAAAT<br>TCAACTCCAGGCAGCAGTAGGGGAACTTCTCCTGCTAGAATGGCTGGCAATGGCG<br>GTGATGCTGCTCTTGCTTTGCTGCTGCTTGACAGATTGAACCAGCTTGAGAGCAAA<br>ATGTCTGGTAAAGGCCAACAACAACAAGGCCAAACTGTCACTAAGAAATCTGCTG<br>CTGAGGCTTCTAAGAAGCCTCGGCAAAAACGTACTGCCACTAAAGCATACAATGT<br>AACACAAGCTTTCGGCAGACGTGGTCCAGAACAAACCCAAGGAAATTTTGGGGAC<br>CAGGAACTAATCAGACAAGGAACTGATTACAAACATTGGCCGCAAATTGCACAAT<br>TTGCCCCCAGCGCTTCAGCGTTCTTCGGAATGTCGCGCATTGGCATGGAAGTCACA<br>CCTTCGGGAACGTGGTTGACCTACACAGGTGCCATCAAATTGGATGACAAAGATCC<br>AAATTTCAAAGATCAAGTCATTTTGCTGAATAAGCATATTGACGCATACAAAACAT<br>TCCCACCAACAGAGCCTAAAAAGGACAAAAAGAAGAAGGCTGATGAAACTCAAG<br>CCTTACCGCAGAGACAGAAGAAACAGCAAACTGTGACTCTTCTTCCTGCTGCAGAT<br>TTGGATGATTTCTCCAAACAATTGCAACAATCCATGAGCAGTGCTGACTCAACTCA<br>GGCCTAA
RSV<br>RNA target	GAAATTAATACGACTCACTATAGGAATTTGATAAGTGCTATTTAAGTCTAACCTTTT<br>TAATCAGAAATGGGGTGCAATTCACT-<br>GAGCATGATAAAGGTTAGATTACAAAATTTGTTTGATAATGACGAAGTAGCATTGT<br>TAAAAATAACATGTTATACTGACAAATTAATTCTTCTGACTAATGCATTAGCCAAA<br>GCAACAATACATACAATTAAATTAAACGGCATAGTTTTTATACATGTTATAACAAG<br>CAGTGAAGTGTGCCCTGATAACAATATTGTAGTGAAATCTAACTTTACAACAATGC<br>CAATATTACAAAATGGAGGATACATATGGGAATTGATTGAATTGACACACTGCTCT<br>CAATTAAATGGTCTAATAGATGACAATTGTGAAATCAAATTTTCTAAAAGACTAAG<br>TGACTCAGTAATGACTGATTATATGAATCAAATATCTGATTTACTTGGGCTTGATCT<br>CCATTCATGAATTATGTTTAGTCTAATTCAATAGACATGTGTTTATTACCATTTTAG<br>TTAATATAAAACCTCATCAAAGGGAAATGGGGCAAATAAACTCACCCAATCAATC<br>AAACCATGAGCACTACAAACGACAACACCACCATG
HRV<br>RNA target	GAAATTAATACGACTCACTATAGAGTGGTGTTTAGTACAAGCACTTCTGTTTCCCC<br>GGAGCGAGGTATAGGCTGTACCCACTGC-<br>CAAAAGCCTTTAACCGTTATCCGCCAACCAACTACGTAAAAGCTAGTAACATCATG<br>TTTCTAAATCGGCGTTCGATCAGGTGGATTTCCCCTCCACTAGTTTGGTCGATGAGG<br>CTAGGAATTCCCCACGGGTGACCGTGTCCTAGCCTGCGTGGCGGCCAACCCAGCGT<br>ATGCTGGGACGCCTTTTTACAGACATGGTGCGAAGACTCGCATGTGCTTGGTTGTG<br>AGTCCTCCGGCCCCTGAATGCGGCTAACCTTAACCCTGGAGCCTTGCGTCACAATC<br>CAGTGATGGTAAGGTCGTAATGAGCAATTCCGGGATGGGACCGACTACTTTGGGTG

	TCCGTGTTTCTCATTTTCTTATTATTGTCTTATGGTCACAGCATATATATTGTATAT ACTGTGATCATGGGCGCTCAAGTATCAACACAGAAGAGTGGATCTCACGAGAATC AGAACATATTAACTAATGGATCCAATCAAACCTTTACGGTTATTAATTATTACAAG GATGCAGCAAGTTCATCTTCAGCCGGGCAATCT
2577-m6A	CUUAAUGUUUUUGCAUUGGm6ACUUUGAGUUAAGAUUAUUUU
2577-A	CUUAAUGUUUUUGCAUUGGACUUUGAGUUAAGAUUAUUUU

Table S2. Comparison with one-pot RNA detection

Method	Nuclease	Amplification	LOD	Time	Reagent stability	Multiplex	Controllable	Refs
EXTRA-CRISPR	Cas12a	RCA	1.35 fM	20 min - 3 h	Low	no	no	1
SURVEY	Cas12	RT-RPA	0.6 copies/μL	15-20 min	Low	no	yes	2
TWI-RAA-Cas13a-Apt	Cas13a	RAA	4 copies	40 min	Low	no	no	3
COP	CpAgo	EXPAR	1 zM	30 min	High	yes	no	4
hybrid Cas system	hybrid Cas	RT-RPA	10 copies	N.A.	Low	yes	no	5
CRISPR-circuit	Cas13a	T7 transcription	aM	15 min	Low	yes	no	6
SATCAS	Cas13a	SAT with T7 transcription	2.41 copies/40 μ L ； 1 aM	40 min	Low	no	no	7
CasTDR$_{1pot}$	Cas13a	RCA	0.1 aM; 1.99 copies/μL	30 min	Low	no	no	8
This work	pfago	RCA	1.13 fM; 1.27copies/μL	1.5h	High	yes	yes	This work

(1) Yan, H.; Wen, Y.; Tian, Z.; Hart, N.; Han, S.; Hughes, S. J.; Zeng, Y. *Nat. Biomed. Eng.* **2023**, *7*, 1583-1601.

(2) Cheng, Z. H.; Luo, X. Y.; Yu, S. S.; Min, D.; Zhang, S. X.; Li, X. F.; Chen, J. J.; Liu, D. F.; Yu, H. Q. *Nat. Commun.* **2025**, *16*, 1166.

(3) Wang, X.; Deng, X.; Zhang, Y.; Dong, W.; Rao, Q.; Huang, Q.; Tang, F.; Shen, R.; Xu, H.; Jin, Z.; Tang, Y.; Du, D. *Biosens. Bioelectron.* **2024**, *257*, 116268.

(4) Lin, Q.; Cao, Y.; Han, G.; Sun, W.; Weng, W.; Chen, H.; Wang, H.; Kong, J. *Anal. Chem.* **2023**, *95*, 13401-13406.

(5) Guk, K.; Yi, S.; Kim, H.; Bae, Y.; Yong, D.; Kim, S.; Lee, K. S.; Lim, E. K.; Kang, T.; Jung, J. *Biosens. Bioelectron.* **2023**, *219*, 114819.

(6) Zhang, Y.; Su, R.; Zhang, Z.; Jiang, Y.; Miao, Y.; Zhou, S.; Ji, M.; Hsu, C. W.; Xu, H.; Li, Z.; Wang, G. *Biosens. Bioelectron.* **2025**, *274*, 117212.

(7) Wang, T.; Bai, L.; Wang, G.; Han, J.; Wu, L.; Chen, X.; Zhang, H.; Feng, J.; Wang, Y.; Wang, R.; Zhang, X. *Biosens. Bioelectron.* **2024**, *263*, 116636.

(8) Li, Y.; Xu, R.; Quan, F.; Wu, Y.; Wu, Y.; Zhang, Y.; Liang, Y.; Zhang, Z.; Gao, H.; Deng, R.; Zhang, K.; Li,

J. *EBioMedicine.* **2025**, *112*, 105564.
